# Association Between Substance Use Behaviors, Developmental Assets and Mental Health: A Glance at Latin American Young College Students

**DOI:** 10.3389/fpsyg.2021.639578

**Published:** 2021-02-25

**Authors:** Denisse Manrique-Millones, Nora Wiium, Claudia Pineda-Marín, Manuel Fernández-Arata, Diego Alfonso-Murcia, José Luis López-Martínez, Rosa Millones-Rivalles

**Affiliations:** ^1^Faculty of Communication Sciences, Tourism and Psychology, Research Institute of Psychology, Universidad San Martín de Porres, Lima, Peru; ^2^Department of Psychosocial Science, Faculty of Psychology, University of Bergen, Bergen, Norway; ^3^Department of Psychology, Fundación Universitaria Konrad Lorenz, Bogotá, Colombia; ^4^Institute of Scientific Research, Universidad de Lima, Lima, Peru

**Keywords:** developmental assets, risk behaviors, young people, well-being, Colombia, Peru

## Abstract

Positive Youth Development (PYD) is an approach that promotes resilience and focuses on youth strengths rather than their weaknesses as done by the traditional deficit-based perspective. Research in Europe and North America show that developmental assets are associated with school success, psychological well-being, and lower health risks among youth and young adults. However, not much research has been done on these associations in Latin American contexts. The purpose of this research study is to assess the association between substance use behaviors, such as drunkenness and the use of illicit drugs, and mental health, together with the mediating role of developmental assets representing youth strengths (e.g., social competence) and contextual resources (e.g., social support at home or school). Cross-national data was collected from Colombian (*n* = 210; 70.4% females) and Peruvian *(n* = 349; 66.5% females) 1st year university students. Results shed light on the protective role of developmental assets regarding substance use behaviors and mental well-being. Specifically, the results showed direct negative associations between developmental assets and substance use behaviors and positive associations of developmental assets with mental health indicators. Internal assets appear to be a stronger predictor of social, emotional, and psychological well-being compared to external assets. We did not find any statistical significance in the association of substance use behavior and mental health. We discuss implications regarding research, policy, and practice.

## Introduction

Positive Youth Development (PYD), a conceptualization that emerged in the 1990's, is a strength-based perspective, which focuses on the potential of youth and young adults (Larson, 2000; Damon, 2004; Lerner, 2017). This positive perspective supersedes the traditional problem-centered model, which provides an unfavorable image of adolescents as mainly characterized by mood disruptions, conflicts with parents, and risk behaviors (Buchanan et al., 1992; Offer and Schonert-Reichl, 1992). While the deficit model draws attention to the weaknesses and risk-taking behaviors of young people and the associated personal and societal consequences, PYD paints a picture of adolescence being a stage of great plasticity and developmental potential, as long as youth and young adults are provided with opportunities and healthy relationships in their immediate environment (Lerner R. M., et al., 2015).

Consistent with PYD, prevention is not synonymous with promotion. Accordingly, a nourishing adolescence and a satisfactory transition to adulthood entails more than just the absence of maladaptive or disruptive behaviors, such as violent behaviors and drug use, as indicated by the deficit model. Rather, positive development requires young people's attainment of a series of evolutionary achievements and sufficient opportunities (Benson et al., 2004). Thus, PYD adopts a perspective that focuses on thriving, well-being, and the competences needed to succeed in social, academic, and professional life.

### Positive Youth Development and Developmental Assets

One core construct within PYD is developmental assets. These assets refer to resources that can be found in the personal, family, peer, school, and community contexts of young people, and that provide support and experiences, which, over the course of time, promote healthy growth and stimulate youth and young adults thriving (Benson, 1997; Benson et al., 1999; Scales and Leffert, 1999; Search Institute, 2007). The assets comprise two dimensions, external assets, which focus on positive experiences, opportunities, and relationships youth need across different aspects of their lives, and internal assets, which refer to the personal skills and positive values of youth and young adults. In line with Benson (2007) developmental asset framework, external assets consist of four categories: *Support* (e.g., caring school and/or neighborhood climate, family support), *Empowerment* (e.g., community values youth, service to others in the community), *Boundaries and expectations* (e.g., family, school boundaries, and high expectations), and *Constructive use of time* (e.g., youth programs, creative activities). Similarly, internal assets consist of four categories: *Commitment to learning* (e.g., school engagement, achievement motivation), *Positive values* (e.g., equality and social justice, honesty), *Social competencies* (e.g., interpersonal and cultural competence, peaceful conflict solution), and *Positive identity* (e.g., sense of purpose, positive view of personal future).

Research on PYD and developmental assets has predominantly involved youth samples from North America (Benson, 1990; Lerner, 2015; YouthPower Learning, 2017), although recent research involving non-American samples is increasing (Soares et al., 2019; Wiium and Dimitrova, 2019). In one of the most representative and largest studies conducted in the United States, where over 99,000 teenagers participated, Benson et al. (1998) concluded that young people who experience more developmental assets also tend to engage less in risk behaviors, such as alcohol misuse, illicit sex, drug, and tobacco use. The authors highlighted that addressing these risk behaviors would require the collaboration of parents, school, and the community.

PYD-related research outside the American context has involved samples from, for example, Western Europe (Matos et al., 2018; Matos M. G., et al., 2018; Wiium et al., 2018; Gómez-Baya and Benítez-Montagut, 2019), Asia (Chen et al., 2016, 2017; Chen and Han, 2017), and Africa (Adams et al., 2018). Despite the increasing interest in PYD, contexts, such as Latin American has barely featured in this research. Thus, the objective of the present study is to examine the concept of developmental assets and the association with substance use behaviors and mental health in two understudied Latin American settings: Colombia and Peru.

### Developmental Assets, Risk Behaviors, and Mental Health

The fundamental role of developmental assets in the promotion of positive youth developmental outcomes has been validated in several cross-cultural studies (Benson, 2003; Whiting et al., 2012). These studies reflected on the need to support a developmental asset approach in which resilience, positive health, and well-being could be developed and promoted by boosting a positive school environment or fostering adequate channels of communication with family and friends. They concluded that support, preservation, and generation of individual well-being and health should be given prominence. Indeed, research studies have associated developmental assets with positive health indicators, and other developmental outcomes in young people. Scales et al. (2000) showed, in a large-scale study conducted in the USA, a relationship of direct proportion between number of developmental assets and success indicators such as leadership, good health, and education outcome.

In addition, a number of empirical studies has confirmed that an increase in the exposure of development assets in young people is associated with a reduction in their involvement in risk behaviors, thus causing them to flourish more (Benson, 1997; Leffert et al., 1998; Scales et al., 2000). Min et al. (2018) reported in a sample of young African American from disadvantaged socioeconomic status that girls with low developmental assets presented more mental health problems, such as depression. The authors argued that primary intervention should be given by strengthening family dynamics and fundamental social systems in school and peers. In a related study, Benson and Scales (2009) looked into how developmental assets are able to mitigate risk behaviors and violence by analyzing several databases of young American students. They concluded that the mechanism by which developmental well-being is promoted is 3-fold: first, through *relationships*, which exposes young people to peers and adults who model socially responsible behavior, second, through *opportunities*, which allows young people to connect and develop relationships with which they can develop constructive social skills and third, through *skills*, which is the development of their unique talents and capabilities via connections to social institutions, such as educational system or youth organizations that promote positive values. This sheds light on the importance of building assets in young people as an approach to reducing risk behaviors and violence. Kia-Keating et al. (2011) in an effort to understand adolescent health development, proposed an integrative model in which, among other foundation principles, they stated that risk and positive outcomes can be mediated by protection and support from significant others. In their model, developmental assets were considered as a potential protector factor in the link between several risk behaviors and mental health, where individuals with substance use disorders have higher probabilities to develop mental health problems, such as depression if they did not experience any developmental assets (Grant et al., 2004; Jane-Llopis and Matytsina, 2006).

Among the few studies found in Latin America, Dutra-Thomé et al. (2018) conducted a study about promotive (i.e., family and community connectedness) and risk factors (i.e., prejudice, exposure to domestic violence) with self-concepts (i.e., self-esteem, self-efficacy) and behavioral problems (i.e., risky sexual behavior, antisocial behavior) among Brazilian young adults. Their results shed light on the important role of family as a promotive factor, specifically family connectedness that was positively linked to self-esteem, whereas neighborhood problems (i.e., poverty and unemployment) was a risk factor for internalizing symptoms. In addition, Hernández-Holguín et al. (2016) used the PYD approach as a basis for analysis in the context of work with youth and young adults in Medellín-Colombia. They observed that aspects, such as resilience, and interactions with the social and family context are fundamental to the understanding of young people's personal development and they tend to influence social transformations through the creation of public policies or social intervention programs. Frías and Barrios (2016) agreed on the benefits of taking the PYD approach as a basis for working with the Mexican population. Using a quantitative methodology, they offered an understanding of youth development, analyzing aspects, such as resources at the social and economic level, and their relationship with PYD.

Not only do developmental assets prevent or reduce risk behaviors among youth and young adults, but they have also been found to enhance and strengthen mental health (Bleck and DeBate, 2016). In a longitudinal study, Bleck and DeBate (2016) reported a negative link between developmental assets in adolescents in 7th through 12th grades and substance use at ages from 24 to 32 years old. Their finding suggests that nurturing and cultivating assets at a young age can have positive lifelong implications. Similarly, in a research study by Hawkins et al. (2008), broad significant effects of assets on mental health were found concluding that an asset-building paradigm during elementary grades resulted in long-term benefits in adulthood.

Notwithstanding, the optimism that these studies can create, they have been carried out mostly in the USA, and the link between assets and youth outcomes may vary depending on cultural and/or geographical context (Adams et al., 2018; Eichas et al., 2019; Soares et al., 2019). Contexts provide different relevant assets, thus potentially resulting in different outcomes. It is important to extend this research to understudied settings, such as Latin American.

### Latin America: Risk Behaviors in the Colombian and Peruvian Contexts

Latin America stands out for its great diversity on biological, geological, and climatological levels, since it embraces a large variety of flora and fauna, as well as multiple climates alongside a vast stretch of natural resources. Latin American countries have made major strides in reducing their extreme poverty indices, one of the cancers of the region, but they are still permeated by many drawbacks, such as inequality as well as child and youth violence that deprives and holds back sustained progress, threatening societal well-being and impeding the social development of this side of the continent.

One major consequence of this extraordinary social disparity and inequality, compromising more young people is the high rate of violence in Latin America (Imbusch et al., 2011).

This is the region of the world that presents the highest rates of armed violence, in 2016, more than 400 homicides per day were reported (Business Insider, 2019). The increase in crime and violence has imposed important social costs and has made socio-economic development processes, democratic consolidation, and regional integration much more difficult in Latin America.

Colombia and Peru are neighboring countries located in the north and western side of South America, respectively. Colombia is a country that has gone through a long period of violence. For almost 60 years, the civilian population suffered the most while caught under the crossfire among “guerrillas,” paramilitary groups and drug traffickers (National Center of Historical Memory, 2013). According to the Ministry of Health (2018), 41% of young people between the ages of 18 and 24 have suffered psychological, sexual, or physical violence. These forms of violence were mainly exercised in the family context, with physical violence being the most commonly used against men and psychological violence against women (National Center of Historical Memory, 2013).

Peru is a country that has met sustainable development over the course of the last 15 years, having successfully reduced poverty, which in turn had other positive consequences, such as the reduction of chronic malnutrition rates. However, Peru has challenges that must be addressed. One important issue that has a big impact on Peruvian youth is violence. Based on the National Institute of Statistics and Informatics and the National Survey on Social Relations (INEI–ENARES, 2018), 81.3% of adolescents from 12 to 17 years of age were victims of psychological or physical violence, making them a vulnerable group.

Colombian proposals to promote the course of youth began in 1986 due to concern over the problems associated with drug trafficking, thus creating a national drug prevention plan.

In 1994, the Ministry of Youth was created, opening spaces dedicated to the socialization of young people and in 1998 a National Plan for the Development of Change was proposed to build better opportunities for education, work, and culture for young people (OMS-Colombia, 2001). As recently as of 2018, the National Development Plan was promulgated, scheduling a place dedicated to Colombian youth and young adults. The main objectives are to promote youth employment and ventures with a transformative vocation for the living conditions of young people and their communities. Likewise, there is the aim to boost youth talent through knowledge transfer on global production trends and challenges and to implement a strategy to support the construction of the youth life project from childhood (DNP, 2018).

Regarding youth policy in Peru, the first National Youth Plan was approved in the period 2006–2011, where three articulating axes were recognized: governance, access, and quality associated with youth development. Unfortunately, the subsequent administration did not follow up with the plan, since it was a policy instrument identified with the previous management and thus, it expired without being implemented. In subsequent years, the Ministry of Education took responsibility for initiative in the youth area, creating the National Youth Secretariat (OECD, 2017). It is only in 2019 that the Government published the National Youth Policy, which establishes a series of objectives to guarantee the integral development of young people. The core objectives are: (a) to develop competencies in the educational process, (b) to increase the access of young people to decent work, (c) to increase comprehensive health care for the young population, (d) to reduce victimization in the young population, (e) to reduce discrimination against young people in vulnerable situations, (f) to increase citizen participation of the young population (El Peruano, 2019).

Colombia and Peru do not only share a culturally diverse background in which indigenous communities were colonized by Spanish conquerors; both are considered megadiverse countries in biodiversity, and they have in common a history of fight against terrorism, guerrillas, and drug cartel. These conditions offer the opportunity and necessity to implement approaches, such as the PYD framework to enhance health and thriving among the most vulnerable: young people.

### The Present Study

The main objective of this research study is to analyze the association of developmental assets (internal and external), mental health indicators (i.e., emotional, social, and psychological well-being) and substance use behaviors (i.e., being drunk, sniffing, inhaling substances, or using other illicit drugs) in two Latin American contexts: Colombia and Peru.

We hypothesize:

- A negative direct effect of substance use behaviors on developmental assets (internal and external).
- An inversely proportional association between substance use behaviors and mental health (emotional, social, and psychological well-being).
- A positive direct effect of developmental assets on mental health.

Besides, judging from earlier studies that have investigated the role of protective factors on risk behaviors, we expect an indirect or mediational effect of developmental assets on the association between substance use behavior and mental health.

Although Colombian and Peruvian youth and young adults have been inevitably affected by extensive socio-political disputes and hostilities in their countries, evidence shows that emerging adults can be prolific and play an important and constructive role in the society (Cunningham et al., 2008). PYD framework can be seen as an opportunity to cultivate resilience and enhance mental health and thriving in Colombian and Peruvian college students.

## Materials and Methods

### Participants

Cross-sectional data were collected from 559 university students, 210 (70.4% females) from Colombia and 349 (66.5% females) from Peru. Participants' age ranged from 18 to 29 years (*M*_*ag*e_ = 19.96, *SD* = 2.70) in Colombia and from 17 to 30 years (*M*_*ag*e_ = 21.64, *SD* = 2.92) in Peru. Table 1 contains information on a number of sociodemographic variables (i.e., gender, ethnic background, socioeconomic status, etc.) and a set of self-report measures per country.

**Table 1:** Descriptive statistics and reliability analysis of study variables across countries.

	**Colombia**	**Peru**
	**(*n* = 210)**	**(*n* = 334)**
Age, range	18–29	17–30
Gender, % females	70.4	66.5
Father's education, % more than high school	58.9	45.6
Mother's education, % more than high school	65.2	43.8
**Developmental assets^α^**		
Commitment	0.71	0.84
Positive values	0.73	0.75
Social competencies	0.68	0.73
Positive identity	0.80	0.78
Support	0.77	0.77
Empowerment	0.66	0.74
Boundaries and expectations	0.67	0.79
Constructive use of time	0.52	0.53
**Mental Health**		
Emotional	0.84	0.85
Social	0.80	0.83
Psychological	0.90	0.89

α *Cronbach's alpha*.

### Measures

*Developmental Assets* (Search Institute, 2007) are personal, family, school, or community resources that provide the support and experiences necessary for promoting positive development throughout the adolescence and youth span. The instrument does not only represent Peter Benson's original 40 assets (1990, 1999, 2007) but goes further to address assets experienced in specific environments (e.g., family and school). A total of 58 items were assigned to two main dimensions: External assets, which refer to family, school, or community characteristics in which the youth lives, and how he or she perceives the existence of supports and limits. External assets are divided into four scales: *Support* (e.g., “I ask my parents for advice”), *Empowerment* (e.g., “I feel valued and appreciated by others”), *Boundaries and expectations* (e.g., “I have friends who set good examples for me”), and *Constructive use of time* (e.g., “I spend time every week in sports, hobby clubs, or organization at school or my community”). Internal assets are the second dimension of developmental assets and imply psychological and behavioral attributes of the person. These assets also consist of four scales: *Commitment to learning* (e.g., “I am eager to do well at school”), *Positive values* (e.g., “I think it is important to help other people”), *Social competencies* (e.g., “I build friendships with other people”), and *Positive identity* (e.g., “I feel good about myself”). Participants were asked to rate the frequency of each asset item on a 4-point Likert scale ranging from “Not at all or rarely” to “Extremely or Almost always.” For the external assets dimension, Cronbach's Alpha was acceptable, ranging from 0.52 to 0.69 and for the internal assets, Cronbach's Alpha ranged from 0.68 to 0.84. For detailed information on the reliability indices per asset category, see Table 1.

*Substance use Behaviors* (Search Institute, 2016) consists of three items (i.e., “Have you been drunk once or more in the last 30 days?,” “Have you sniffed or inhaled substances to get high once or more in the last 12 months?,” and “Have you used other illicit drugs (e.g., cocaine, LSD, heroin, amphetamines, etc.) once or more in the last 12 months?.” Responses were rated on a binary scale, 0 (No), and 1 (Yes). For data analysis, a composite score, reflecting the number of substance use behaviors engaged in was created from the three items, with scores ranging from 0, where the individual did not engage in any of the risk behaviors to 3, where the individual engaged in all three risk behaviors.

*Mental Health Continuum* (MHC-SF*;* Keyes, 2005). The MHC-SF consisted of 14 scale items that measures well-being. It is divided in three dimensions: Emotional well-being (three items, e.g., “interested in life”), social well-being (five items; e.g., “That you had warm and trusting relationships with others”) and psychological well-being (six items; e.g., “Confident to think or express your own ideas and opinions”). Participants rated the frequency of their answers in the last month on a 6-point Likert scale, ranging from “Never” to “every day.” In the present study, Cronbachs'Alphas of the three mental health indicators ranged from 0.84 to 0.90 for Colombia and 0.83 to 0.89 for Peru.

### Procedure

The present research forms part of a large-scale cross-sectional study on PYD across countries (see Wiium and Dimitrova, 2019). An institutional review board in Colombia (i.e., The Bioethics Committee of the Konrad Lorenz University Foundation) and in Perú (i.e., The Research Committee of the Universidad San Martín de Porres), provided ethical clearance for conducting the research study in both countries, Colombia, and Peru.

The survey was translated from English to Spanish and back-translated to assure linguistic equivalence of the instrument. A set of self-administered scales and socio-demographic information was completed by young people using two approaches, physically (i.e., paper and pencil) in which most youngsters were recruited from universities, and remotely (i.e., online platform) in which a link was enabled and disseminated in different social media sites. In both countries, informed consent was requested in which participants could withdraw from the survey at any time. Participants were also assured that the results were to be used solely for research purposes and that their participation was anonymous.

### Data Analysis

Missing cases on study variables were 2% or less. In descriptive analysis, these cases were handled using pairwise deletion. Descriptive analyses on study variables as well as reliability tests on composite variables were undertaken to assess the distribution of study variables and the internal consistencies of items measuring the developmental asset categories and mental health indicators. A composite score reflecting the number of substance use behaviors engaged in was created and used in the analyses. For the asset categories, composite variables that indicated the number of assets for each asset category were created by first recoding the 4-point Likert scale into a binary one, where response alternatives 1 and 2 were recoded as asset not present, and 3 and 4 recoded as asset present. Mean scores were created for the mental health indicators in descriptive analysis.

To assess the structural relations among substance use behaviors, developmental assets and mental health, measurement invariance (i.e., configural invariance, metric invariance, and scalar invariance) across country and gender was first examined by conducting a set of Multigroup Confirmatory Factor Analyses (MGCFA) on the items measuring developmental assets and mental health, with Mplus (Muthén and Muthén, 1998–2017). Configural invariance is when the asset categories load onto the latent factor (internal assets or external assets) in the same manner across groups (i.e., country and gender). For metric invariance, the factor loadings of the asset categories on internal and external assets are the same across groups, while for scalar invariance, it is not only the factor loadings that are identical but the intercepts as well. In MGCFA, the eight asset categories (four for internal and the other four for external) were treated as observed variables to determine how they each load onto their underlying latent factor. For mental health, a three-factor model was estimated in MGCFA, where individual observed items loaded onto the emotional, social and psychological indicators of mental health.

After measurement invariance was assessed, a series of structural equation modeling: (1) for each country, (2) an unconstrained model, and (3) a constrained model was conducted to ascertain the best-fit model. Gender, age, and religion along with father and mother's educational background were treated as covariates. Chi-square tests and fit indices, such as the Tucker Lewis Index (TLI; acceptable >0.90), the Root Mean Square Error of Approximation (RMSEA; acceptable below 0.08), and Comparative Fit Index (CFI; acceptable above 0.90) (Hu and Bentler, 1999; Byrne, 2008) were used to evaluate model fit in both measurement and structural equation models. All analyses in Mplus were conducted using the Maximum likelihood estimation with robust standard errors to handle missing cases.

## Results

### Descriptive and Correlation Analyses

In findings not presented in tables, 73.7% of the total sample did not engage in any of the three substance use behaviors, while 19.1% engaged in one, 4.6% in two, and 2.6% in all three substance use behaviors. In addition, the number of assets reported was above average for all the asset categories except for *Constructive use of time*. The responses to how much participants have experienced the different mental health indicators were mostly between “2–3 times a week” and “almost every day” (Table 2). The variable indicating the number of substance use behaviors engaged in correlated negatively with all four internal assets: *Commitment to learning, Positive values, Social Competence*, and *Positive identity*, with correlations (weak) ranging from *0.09* to −*0.24*, and positively with one external asset: *Constructive use of time* (*r* = 0.13, *p* < 0.01). In addition, the substance use behaviors variable correlated negatively with one of the mental health indicators: (*r* = −0.10, *p* < 0.05). Moreover, positive correlations (weak to moderate) ranging from 0.10 to 0.51 were observed between the developmental asset categories and the mental health indicators (Table 2).

**Table 2:** Descriptive and correlation analysis of demographics, developmental assets, and mental health indicators for the total sample.

**Study variables**		**2**	**3**	**4**	**5**	**6**	**7**	**8**	**9**	**10**	**11**	**12**	**13**	**14**	**15**	**16**	**17**	**18**
1. Country^a^		−0.04	0.28^**^	−0.13^**^	−0.21^**^	0.28^**^	−0.25^**^	−0.04	−0.10^*^	0.11^**^	0.11^*^	−0.11^**^	−0.20^**^	−0.15^**^	−0.20^**^	−0.01	−0.01	−0.05
2. Gender^b^		–	0.06	−0.04	−0.06	0.14^**^	−0.19^**^	0.21^**^	0.14^**^	0.17^**^	0.05	0.14^**^	0.13^**^	0.17^**^	−0.16^**^	0.07	0.01	0.09^*^
3. Age			–	−0.06	−0.22^**^	0.16^**^	−0.06	0.02	−0.07	0.06	0.06	−0.17^**^	−0.05	−0.10^*^	−0.18^**^	0.05	−0.01	0.07
4. Father's education				–	0.39^**^	−0.12^**^	0.08	−0.09^*^	−0.02	−0.05	−0.07	0.14^**^	0.08	0.04	0.12^**^	−0.10^*^	0.05	−0.03
5. Mother's education					–	−0.21^**^	0.12^**^	−0.04	0.03	−0.04	−0.10^*^	0.11^*^	0.11^*^	0.07	0.17^**^	−0.12^**^	0.01	−0.09^*^
6. Religion						–	−0.15^**^	0.10^*^	0.12^**^	0.13^**^	0.11^**^	0.07	0.03	0.07	0.01	0.13^**^	0.12^**^	0.11^**^
7. Risk behaviors							–	−0.17^**^	−0.16^**^	−0.24^**^	−0.09^*^	−0.03	−0.01	−0.05	0.13^**^	−0.08	−0.05	−0.10^*^
8. Commitment to learning								–	0.43^**^	0.38^**^	0.36^**^	0.28^**^	0.37^**^	0.42^**^	0.01	0.31^**^	0.23^**^	0.38^**^
9. Positive values									–	0.57^**^	0.38^**^	0.35^**^	0.36^**^	0.36^**^	0.19^**^	0.35^**^	0.44^**^	0.46^**^
10. Social competence										–	0.47^**^	0.31^**^	0.33^**^	0.32^**^	0.07	0.37^**^	0.40^**^	0.50^**^
11. Positive identity											–	0.24^**^	0.33^**^	0.28^**^	-0.00	0.43^**^	0.39^**^	0.51^**^
12. Support												–	0.52^**^	0.62^**^	0.24^**^	0.33^**^	0.32^**^	0.34^**^
13. Empowerment													–	0.60^**^	0.23^**^	0.36^**^	0.36^**^	0.39^**^
14. Boundaries and expectations														–	0.14^**^	0.32^**^	0.31^**^	0.32^**^
15. Constructive use of time															–	0.12^**^	0.18^**^	0.10^*^
16. Mental health–emotional																–	0.53^**^	0.72^**^
17. Mental health–social																	–	0.59^**^
18. Mental health–psychological																		–
Descriptive analysis	Range	1–2	15–29	1–2	1–2	1–5	0–3	0–7	0–11	0–8	0–6	0–7	0–6	0–9	0–4	1–6	1–6	1–6
	Mean	1.68	20.99	1.51	1.52	3.80	0.36	6.27	7.63	6.10	4.19	4.31	4.69	6.86	1.29	4.61	3.45	4.56
	S.D.	0.47	2.95	0.50	0.50	1.14	0.69	1.15	2.07	1.81	1.72	1.78	1.38	1.76	1.14	0.93	1.14	0.98

* *p < 0.05*.

** *p < 0.01. S.D., Standard deviation*.

a *Country: (1) Colombia and (2) Peru*.

b *Gender: (1) Male and (2) Female*.

### Measurement and Structural Invariance Models

In a series of MGCFA conducted for the developmental assets to assess measurement invariance (configural, metric, and scalar), partial scalar invariance across country and gender was established for the developmental assets, where based on results from modification indices, the equality rule was relaxed for the intercept of *Positive values, Social competences*, and *Constructive use of time*. Although *Constructive use of time* loaded poorly onto the latent external asset factor, we kept the variable in the analyses as removing it did not cause any significant changes to the findings. Furthermore, in MGCFA of the mental health indicators, scalar invariance was established (Table 3). Thus, meaningful comparison of the developmental assets and mental health can be made across country and gender, albeit with caution for the assets.

**Table 3:** Measurement invariance models for developmental assets and mental health indicators by country and gender.

**Model**	**Model fit indices**							
	***χ^*2*^ (df)***	***RMSEA***	***90% CI RMSEA***	***CFI/TLI***	***χ^*2*^ (df)***	***RMSEA***	***90% CI RMSEA***	***CFI/TLI***
**Developmental Assets**								
**Country**					**Gender**			
Configural invariance	49.79 (24)	0.063	0.038–0.088	0.977/0.947	35.06 (24)	0.042	0.000–0.070	0.990/0.976
Metric invariance	51.26 (30)	0.051	0.025–0.074	0.981/0.965	50.85 (30)	0.051	0.025–0.075	0.980/0.963
Scalar invariance	99.82 (36)	0.081	0.062–0.100	0.943/0.912	85.52 (36)	0.072	0.052–0.092	0.953/0.927
*Partial scalar invariance*	*62.07 (33)*	*0.057*	*0.034*–*0.078*	*0.974/0.956*	*61.01 (33)*	*0.056*	*0.033*–*0.078*	*0.974/0.955*
**Mental Health**								
Configural invariance	295.97 (126)	0.070	0.060–0.081	0.946/0.921	249.91 (126)	0.061	0.050–0.072	0.959/0.941
Metric invariance	306.85 (137)	0.068	0.057–0.078	0.946/0.928	258.47 (137)	0.058	0.047–0.068	0.960/0.947
*Scalar invariance*	*355.48 (148)*	*0.072*	*0.062*–*0.081*	*0.934/0.918*	*272.85 (148)*	*0.056*	*0.046*–*0.067*	*0.959/0.949*

*χ^2^, Chi-Square; df, degrees of freedom; CFI, Comparative Fit Index; TLI, Tucker Lewis Index; RMSEA, Root Mean Square Error of Approximation; CI, Confidence Interval; Configural, same number of factors and factor loading pattern across groups; Metric, equality of the factor loadings; Scalar, equality of the factor loadings and intercepts*.

Once (partial) scalar invariance was established for the developmental assets and mental health, the association between substance use behaviors and mental health indicators (emotional, social and psychological) as well as possible mediating role of the developmental assets were examined in structural equation modeling. In the first step, the SEM model (i.e., analyzed separately for the two countries) had adequate fit indices for both Peru and Colombia (Table 4). The fit indices of the unconstrained model in the second step were also acceptable, and in the third step, the fit indices of the constrained model were similar to those of the unconstrained model (Table 4). Using the fit indices to evaluate the models, our best fitting model is thus, the constrained model.

**Table 4:** Structural equation modeling of risk behaviors, developmental assets, and mental health.

**Model**	**Model fit indices**			
	***χ^*2*^ (df)***	***RMSEA***	***90% CI RMSEA***	***CFI/TLI***
**Risk behaviors, internal assets, and mental health**				
Single group solutions				
Colombia	324.09 (203)	0.054	0.043–0.065	0.928/0.907
Peru	375.40 (203)	0.051	0.043–0.060	0.936/0.918
Unconstrained	801.48 (434)	0.057	0.051–0.063	0.917/0.900
*Constrained*	*845.67 (467)*	*0.056*	*0.050*–*0.062*	*0.914/0.904*
**Risk behaviors, external assets, and mental health**				
Single group solutions				
Colombia	327.08 (203)	0.055	0.044–0.066	0.924/0.902
Peru	361.04 (203)	0.049	0.041–0.057	0.939/0.922
Unconstrained	773.02 (434)	0.055	0.048–0.061	0.920/0.904
*Constrained*	*808.18 (467)*	*0.053*	*0.047–0.059*	*0.920/0.910*

*χ^2^, Chi-Square; df, degrees of freedom; CFI, Comparative Fit Index; TLI, Tucker Lewis Index; RMSEA, Root Mean Square Error of Approximation; CI, Confidence Interval*.

**Figure 1:**
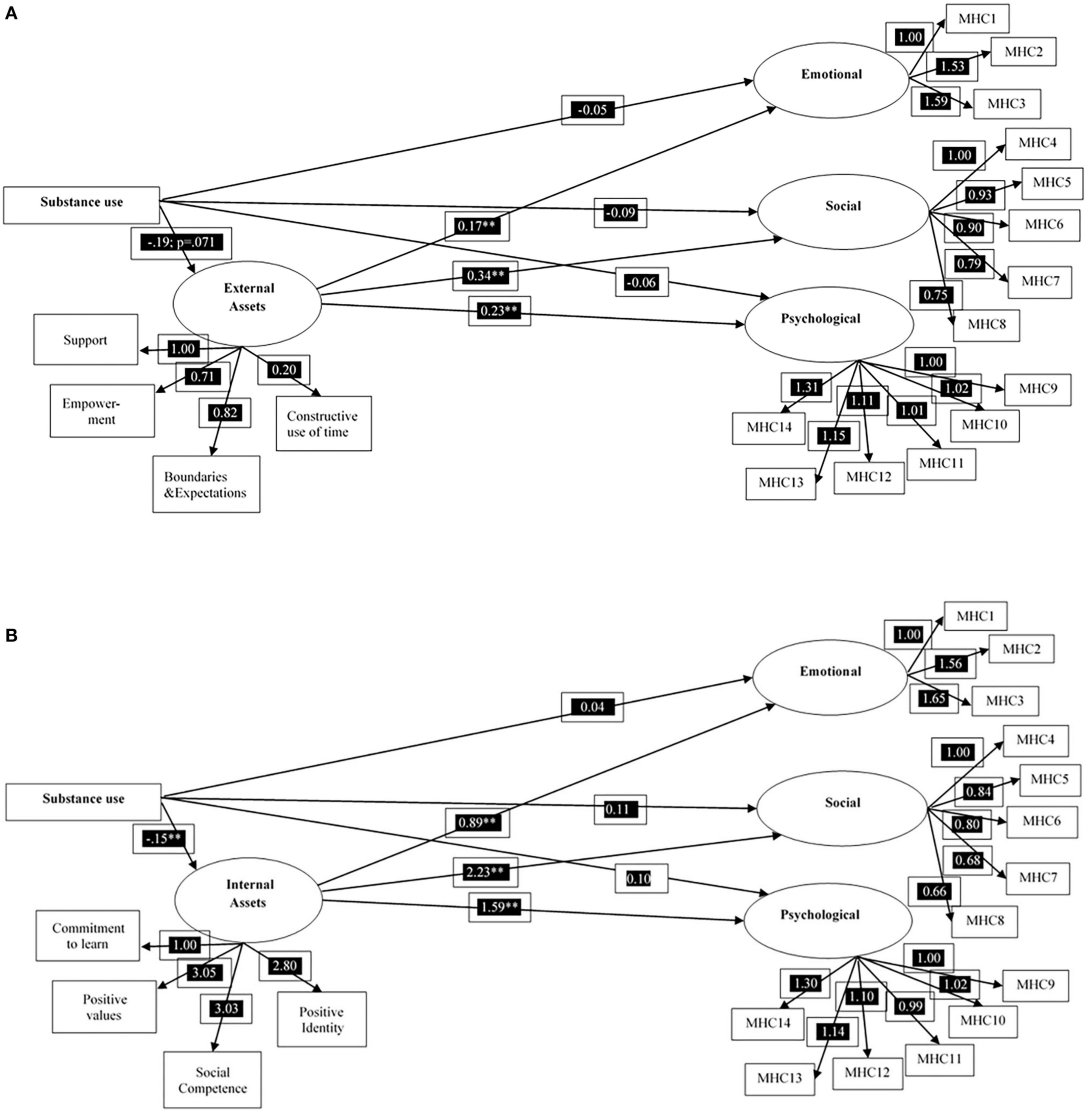
Structural invariance model of substance use behaviors, **(A)** Internal Assets and Mental Health—Unstandardized Coefficients from Mplus SEM Analysis (controlled for age, gender, religion, father, and mother's education) and **(B)** External Assets and Mental Health—Unstandardized Coefficients from Mplus SEM Analysis (controlled for age, gender, religion, father, and mother's education). MHC, Mental Health Continuum; **p < 0.01.

In Figures 1A,B, the results of the constrained models with internal assets and external assets as mediating variables, respectively, are presented. Before the assessment of possible mediating roles of internal and external assets, there was an indication that the substance use behavior variable was negatively associated with the emotional, social and psychological indicators of mental health, although these associations (with age, gender, and parents' educational background controlled for) were not significant. With the inclusion of the mediating role of internal assets, there was still no direct associations between substance use behaviors and mental health. However, the risk behavior variable was significantly and negatively associated with the internal asset's variable (unstandardized coefficient of −0.15 and significant at *p* < 0.01). In addition, the internal assets variable was significantly and positively associated with the emotional, social, and psychological indicators of mental health, with unstandardized coefficients ranging from 0.89 to 2.23, all coefficients significant at *p* < 0.01. Similarly, no direct associations were observed between substance use behaviors and the mental health indicators. Nonetheless, like internal assets, a negative association was observed between substance use behaviors and external assets (−0.19) at borderline significance level (*p* = 0.071). The external assets variable was also significantly and positively associated with the mental health indicators although the associations appear to be weaker compared to those observed for internal assets (unstandardized coefficients ranging from 0.17 to 0.34, all coefficient significant at *p* < 0.01.

## Discussion

The purpose of this research study was to assess the association between substance use behaviors, such as drunkenness and the use of illicit drugs and mental health, together with possible mediating role of developmental assets among young college students living in Colombia and Peru. The results indicated a negative relationship between substance use behavior and developmental assets as well as a positive association of developmental assets on mental health indicators. Internal assets appeared to be a stronger predictor of social, emotional, and psychological well-being relative to external assets. The association between substance use behavior and mental health was not statistically significant.

Our first hypothesis regarding a direct negative association between substance use behaviors and developmental assets was confirmed. As proposed by the PYD framework, developmental assets tend to be protective against risk behaviors, as well as promote positive developmental outcomes (Benson et al., 1998). In our study, college students in Colombia and Peru, who engaged in more risk behaviors were also less likely to report developmental assets, as shown in previous empirical studies (e.g., Scales, 1999). Moreover, Leffert et al. (1998) confirmed that assets associated with positive peer influence were core predictors of reduced substance and alcohol use.

As risk behaviors threaten well-being, they have negative repercussions on health, or at least compromise the development of individuals who engaged in them. Adolescents and young people in Latin America are permanently exposed to different risk behaviors, since the region is known for having a history riddled with violence, crime and the permanent fight against drug trafficking, in which young people are considered as highly vulnerable.

In Peru, since the advent of the 1990's gangs, the dynamics of crime associated with drug trafficking have brought about new stereotypes, such as the delinquent and the youth gang, which became attributed to young people, and made them even more vulnerable. This, as well as the practice of gendered violence (manifested in different forms: psychological, verbal, and physical), has changed the way in which the populations of young people in Peru undertake life projects and development. A Peruvian national study about drug consumption (CEDRO, 2017) showed that the most consumed illegal drug is cannabis, followed by PBC (Cocaine pasta) and cocaine hydrochloride, and to a lesser extent other drugs, such as ecstasy, which is also the most reported illegal drug. Consistent with CEDRO's study, 26 out of 100 people reported having received at least one offer and three out of 10 agreed to try it and did return to consuming it. The problem here is that there is high offer with respect to the demand, and as a result the price is low, which makes the drugs appealing and more accessible for the young population.

The reality of young Colombians has been marked by violence, in different manifestations: forced displacement, sexual violence, kidnapping, homicides, conscription, and mutilations in antipersonnel mines among others, which have left them with indelible physical and psychological consequences (National Center of Historical Memory, 2013; Hewitt et al., 2014). The consumption of legal and illegal substances is also a problem in the reality of young Colombians (Pérez-Gómez et al., 2018). More than 230,000 teenagers started drinking alcohol before the age of 18, and substances, such as tobacco, marijuana, cocaine, and “bazuco” (a cocaine residue) are among the most consumed substances by young Colombians (Ministry of Health Social Protection UNODC, 2014; Córdoba-Paz et al., 2017; Pineda-Marín et al., 2018).

Concerning our second hypothesis on the direct effect of substance use behavior on mental health, we did not observe any statistically significant associations between substance use and the emotional, social and psychological indicators of mental health, although there were some indications of a negative association. A plausible explanation for why the negative impact of substance use behaviors on mental health was not evident in our study can be related to the normalization of such behaviors in the region. Highly violent contexts make young people incorporate risk behaviors into their daily lives and these do not necessarily constitute a great liability compared to other behaviors inherent to the context (Ovallos-Parales and Rojas-Hoyos, 2019). For example, in some cities riding a bike to the university or having a mobile call in the street constitute a great risk (El Comercio, 2020), because one can get robbed and murdered, but smoking or drinking would not be considered as a risk behavior, since actions of self-negligence cannot be internalized as such. Some studies indicate that among the reasons for initiating alcohol consumption in the Latin American region, besides the availability of the substance, are: normalization, favorable attitudes toward consumption, low perception of risk and inadequate regulatory influences (Martínez, 2006; Fagan et al., 2012).

The results lead us to consider the importance of substance abuse prevention education as a right to which all young people should have access. The failure of young people to recognize substance use behaviors or their inability to understand the consequences of such behaviors on mental health, evidence the negligence of governments in social investment, which in the medium and long term, deepens the gap in the development of young people, increases inequality among Latin Americans and in general, maintains the underdevelopment of the nations.

Our third hypothesis was corroborated by the positive direct associations of developmental assets on all three indicators of mental health that were observed. Specifically, both internal and external assets were significant predictors, although the former appeared to be a stronger predictor of emotional, social, and psychological well-being. Thus, Latin American college students who reported more of the developmental assets were also more likely to score higher on the mental health indicators. This result is in line with previous research studies that reported more presence of internal assets over external assets (Scales, 1999; Scales et al., 2000; Wiium, 2017). It would seem that youth development programs in Latin American are not nurturing external assets, which, as much as the internal assets, are of crucial importance for PYD (Benson, 2007).

One possible reason as to why young people did not score high on external assets can be due to the distrust that they have in their public institutions and government. For example, more than 75% of young Peruvians distrust the Executive, Legislative and Judicial systems. Public policies on youth are generally framed within the transitory nature of this life cycle, putting aside long-term programs and focusing on fragmented actions that touch on some aspect of the problem but without articulating other actions equally necessary. Such a situation makes the government lack of commitment toward youth and young adults needs quite evident. In addition to this, there is a widespread feeling among urban-marginal youth that the government is not interested in supporting them, but only the elites. These unfavorable ratings are a challenge for the State, since trust in institutions is essential for social stability and an effective application of public policies and democracy. In a study by the Colombian Ministry of the Interior, results showed that young people think about corruption as the main problem in the country, and show themselves as a critical, disgusted generation who distrusts the institutions of representative democracy. However, they are interested in politics and active citizenship (LAPOP, 2018). This low trust of youth and young adults in government institutions represents a challenge for the State and its impact should be taken seriously, so that young people can feel supported by their authorities and national representatives.

### Limitations and Recommendations

Despite the fact that the present study contributes to the universality of the developmental assets and their positive or protective effect, it is not exempt from limitations. One of them has to do with the nature of the sample, which was carried out in urban areas of the capital of both countries, this may restrict the generalization of the results, although, results are generally in line with previous cross-sectional and longitudinal studies conducted in other contexts. It is still essential to carry out studies with a more representative sample of the entire Latin American region. Likewise, our sample composed mainly of university students, a population that might not readily reflect a vulnerable group, conducive enough to study the effect of resilience. Nevertheless, as mentioned earlier, youth in Colombia and Peru in general, live in vulnerable conditions where they are exposed to violence, drug trafficking, and increasing health compromising risk behaviors.

Second, it might be that some assets, for example *Constructive use of time*, which had quite low factor loading, may not be adequate measures of the assets in Latin American contexts, although reliability coefficients ranged from acceptable to good. Thus, while quantitative methods provide rich and important information in various settings and cultures, in future studies, the use of a mixed method approach can allow other resources and opportunities available to youth and young adults in the Latin American contexts, not appropriately captured in the developmental assets to be explored in the qualitative part of such studies. Third, the responses to the substance use behaviors items were dichotomous but, in the literature, we might encounter other responses alternatives that introduce more variability. In future studies these responses that allow for more variation can be used. Fourth, data was self-reported, and this can lead to either over or under reporting of sensitive information, especially when it relates to drug consumption, crime activities or in general undesired behaviors. Nevertheless, the anonymity and confidential disclosure of our research should not compromise the reliability of the results (Richter et al., 2011).

### Implications for Policy and Practice

Regardless of the limitations, the present findings shed light on the association between substance use behaviors, developmental assets, and mental health in two Latin American settings where little to no prior research has been done. As one may see the higher crime and violence rates of the region as constant challenges and threats that can undermine the positive functioning of the young adults, there is evidence that building assets in the life of emerging adults can contribute to protection against risk behaviors or even suppression of undesired behaviors (Benson et al., 1998; Leffert et al., 1998).

The fragility of young Latin Americans requires that public policies respond with a long-term perspective on the rights of this population. A complete package of interventions in which they could benefit from a set of services necessary for optimal development, instead of a simple aggregation of programs is needed. Extensive multisectoral policies that articulate spaces of well-being, social integration, and participation for sustained growth of their capacities and living conditions are necessary.

In the present study, internal assets were reported more often than external assets. However, it is important to cultivate and nurture equally both asset dimensions. Although some external assets might be seen as difficult to reach because they involve some investment (e.g., youth programs or creative activities), there are others, such as support, in which young people can feel protected or assisted from the warmth of their home, school, or community.

### Conclusions

It has been found in earlier research that within the PYD framework, developmental assets have a truly positive influence on a number of positive outcomes (e.g., academic success, resiliency, and psychological well-being). The present study adds to the PYD literature by bringing to the table some important findings regarding the associations between substance use behaviors, developmental assets, and mental health from the Latin American context. Youth and young adults in the region are believed to have the potential to develop into healthy members of society who contribute to it in a productive manner. Despite their social, and often politically driven woes, their stance on society has evolved to integrate democratic values (Pew Research Centre, 2017), as well as an optimistic outlook on future (OECD, 2017). PYD perspective as a life-governing principle has the potential to change the perception of young Colombians and Peruvians role in society and promote a positive attitude and development.

## Data Availability Statement

The raw data supporting the conclusions of this article will be made available by the authors, without undue reservation.

## Ethics Statement

The studies involving human participants were reviewed and approved by Institutional Review Board in Colombia by The Bioethics Committee of the Konrad Lorenz University Foundation and in Peru by The Research Committee of the Universidad San Martín de Porres. Participants provided their written informed consent to participate in this study.

## Author Contributions

DM-M conceived the idea, development of the study, wrote a first draft of the manuscript, and subsequently contributed to the rewriting and refinements. NW was responsible for the original design providing methodological expertise, conducting data analysis, and giving critical insight revising the manuscript. CP-M, RM-R, DA-M, JL-M, and MF-A were responsible for the data acquisition and made substantial intellectual contributions to the work. All authors have approved the final version of the manuscript and agreed to be accountable for all aspects of their work.

## Conflict of Interest

The authors declare that the research was conducted in the absence of any commercial or financial relationships that could be construed as a potential conflict of interest.
